# A critique of using epitaxial criterion to discriminate between protogenetic and syngenetic mineral inclusions in diamond

**DOI:** 10.1038/s41598-024-59432-6

**Published:** 2024-04-15

**Authors:** Marco Bruno, Stefano Ghignone, Dino Aquilano, Fabrizio Nestola

**Affiliations:** 1 Dipartimento di Scienze della Terra, Università degli Studi di Torino, Via Valperga Caluso 35, 10125 Turin, TO Italy; 2 NIS, Centre for Nanostructured Interfaces and Surfaces, Università degli Studi di Torino, Via G. Quarello 15/a, 10135 Turin, TO Italy; 3 Dipartimento di Geoscienze, Università degli Studi di Padova, Via Gradenigo 6, 35131 Padua, Italy

**Keywords:** Diamond inclusions, Protogenesis, Syngenesis, Epitaxial criterion, Geology, Mineralogy

## Abstract

Distinguishing syngenetic from protogenetic inclusions in natural diamonds is one of the most debated issues in diamond research. Were the minerals that now reside in inclusions in diamonds born before the diamond that hosts them (protogenesis)? Or did they grow simultaneously and by the same reaction (syngenesis)? Once previously published data on periclase [(Mg,Fe)O] and magnesiochromite (MgCr_2_O_4_) inclusions in diamond have been re-analysed, we show that the main arguments reported so far to support syngenesis between diamond and its mineral inclusions, definitely failed. Hence: (a) the epitaxial relationships between diamond and its mineral inclusion should no longer be used to support syngenesis, because only detecting an epitaxy does not tell us which was the nucleation substrate (there are evidences that in case of epitaxy, the inclusion acts as a nucleation substrate); (b) the morphology of the inclusion should no longer be used as well, as inclusions could be protogenetic regardless their shapes. Finally, we advance the hypothesis that the majority of inclusions in diamonds are protogenetic, e.g., they are constituent of rocks in which diamonds were formed and not products of reactions during diamond growth.

## Introduction

Mineral inclusions in diamond might be classified according to the timing of their formation related to the host diamond^1,2^: (i) *syngenetic* inclusions form at the same time as diamond and through the same chemical reaction; (ii) *protogenetic* inclusions are instead pre-existing minerals; (iii) *epigenetic* inclusions are late crystallized phases that grew after diamond formation. A protogenetic inclusion can (partly or fully) re-equilibrate with the diamond-forming medium by intra-crystalline diffusion and/or exchange reactions before being encapsulated, thus its composition could be fully reset during diamond formation.

When analysing the literature about diamonds and their mineral inclusions, it appears that most of the studies assumed a syngenetic relationship; however, syngenesis remains unverified in many cases (if not always), serious doubts may be raised about this strong assumption. Distinguishing between syngenesis and protogenesis is as crucial as it is extremely difficult and controversial^3^. The three most common reported potential indicators of syngenesis are:

1. the imposition of the diamond morphology on the inclusions^1,4–9^ (morphological criterion, hereafter MC);
2. diamond growth zones interrupted by the diamond/inclusion contact (growth zones criterion, hereafter GC)^7^;
3. epitaxial relationships between the inclusion and its host (epitaxial criterion; hereafter EC)^5,6,8–10^.

The MC is based on the belief that diamond can impose its cubo-octahedral morphology upon the inclusion only during latter’s growth^6^. The MC was challenged by several researchers^2,3,11–13^. Nestola et al.^2^ measured the crystallographic orientations of olivine inclusions finding that within a single diamond, several inclusions with both pseudo-cuboctahedral and lobate (i.e., partly resorbed) morphologies have equal crystallographic orientation among them but different orientation with respect to the host. Olivines were interpreted as protogenetic, being relicts of an original monocrystal that underwent dissolution during diamond growth. Same observations were reported on garnet and pyroxene inclusions^12,14,15^. Thus, the discovery of protogenetic inclusions with diamond-imposed morphology contradicts the MC criterion.

The GC is strictly related to the morphological one. According to Bulanova^7^ (see her Fig. 11), a protogenetic inclusion in diamond keeps its own original and non-modified morphology (e.g., diamond growth zones develop around inclusions without altering the diamond/inclusion interfaces); in all other cases, the inclusion is considered syngenetic. This interpretation can no longer be considered valid as many inclusions in diamonds showing imposed cubo-octahedral morphology are protogenetic^2^, therefore the GC must be rejected. Indeed, a mineral inclusion with cubo-octahedral morphology implies a modification of the inclusion/host interface during or after its entrapment in the diamond^16^, thus contradicting the initial hypothesis of the GC, which states that the shape of a protogenetic inclusion is not altered during the diamond growth.

We will show here that also the third criterion (EC) cannot be considered as a reliable argument to demonstrate the syngenetic relationship between diamonds and their mineral inclusions. We will make use of recent experimental observations and some new considerations to correctly interpret the relative crystallographic orientations of mineral inclusions in diamond^2,4,6,10,13–15,17–27^. Especially, we will focus our attention on periclase [(Mg,Fe)O] and magnesiochromite (MgCr_2_O_4_) inclusions, recently considered syngenetic by some researchers on the basis of the EC. We will show that although the EC is satisfied there is evidence that, instead, it could indicate a protogenetic origin. Based on this new interpretation, we will propose an alternative explanation for the formation of periclase and magnesiochromite inclusions showing epitaxial relationships with diamond.

## Epitaxy and epitaxial criterion

Epitaxy occurs when a crystal A (deposit) is formed over a crystal B (substrate) sharing a common lattice interface^28^. Accordingly, an epitaxial interface is generated: (hkl)_A_/(h’k’l’)_B_, where (hkl)_A_ and (h’k’l’)_B_ are the crystal faces in contact. When epitaxy occurs, a specific crystallographic orientation between the two crystal phases sets up, which is usually made explicit by defining either (i) the absolute angle difference (angular misfit) between the main crystallographic axes of phases A and B or (ii) the 2D Lattice Coincidence cells (2D-LCs hereinafter) of the epitaxial (hkl)_A_/(h’k’l’)_B_ interface. A 2D-LC is defined by the coincidences (and the related percent misfits) of two couples of parallel vectors: [uvw]_A_//[uvw]_B_ and [u’v’w’]_A_//[u’v’w’]_B_. Hence, the scalars [uvw]_A_ · [u’v’w’]_A_ and [uvw]_B_ · [u’v’w’]_B_ characterize the 2D cell areas of the (hkl)_A_ and (h’k’l’)_B_ faces, respectively^28^.

A necessary but not sufficient condition to have epitaxy between two crystalline phases A and B is a 2D-LC at the (hkl)_A_/(h’k’l’)_B_ interface^28^: i.e., the smaller the misfit in the area and in the absolute angles of the 2D-LC, the greater the probability of having epitaxy. However, to physically and correctly evaluate the probability to observe an epitaxy between two phases, it is fundamental to calculate the adhesion energy between the phases A and B. The adhesion energy (${\beta }_{adh}^{ A/B}$, expressed in J/m^2^) is the energy recovered when two condensed phases are brought into contact along the A/B interface. Without an estimate of this thermodynamic quantity, the only geometrical description of the 2D-LC does not allow to characterize in detail the epitaxial phenomenon and to evaluate the probability of observing a preferential crystallographic orientation between the two phases.

The EC is based on the analysis of the Crystallographic Orientation Relationships (CORs hereinafter) between inclusion and host (i.e., how the crystallographic axes of the inclusion are arranged with respect to those of the host phase). Four types of CORs can be distinguished^29^: (1) *specific*, (2) *rotational statistical*, (3) *dispersion statistical* and (4) *random*. In specific CORs, at least two crystallographic directions of the inclusion are fixed to the host. In rotational statistical CORs, only one inclusion crystallographic orientation is fixed to that of the host. In dispersion statistical CORs, an inclusion crystallographic direction is not exactly fixed to the host, but is dispersed around it, within a certain misorientation angle range. In all other cases, the inclusion crystallographic directions are randomly oriented relatively to the host.

When specific CORs are individuated, inclusion is often considered syngenetic^3,5–7,19,27^: it is supposed that a mineral nucleates and grows on a diamond face with a specific crystallographic orientation and, subsequently, is embedded by the diamond itself. Unfortunately, this interpretation is almost certainly incorrect. Indeed, the identification of a specific COR between inclusion and host does not provide any information about which phase acted as a substrate for the nucleation of the other. Therefore, specific CORs and epitaxy between diamond and inclusion do not demonstrate syngenesis at all. Thus, also EC must be refused as a proof of syngenesis.

**Figure 1:**
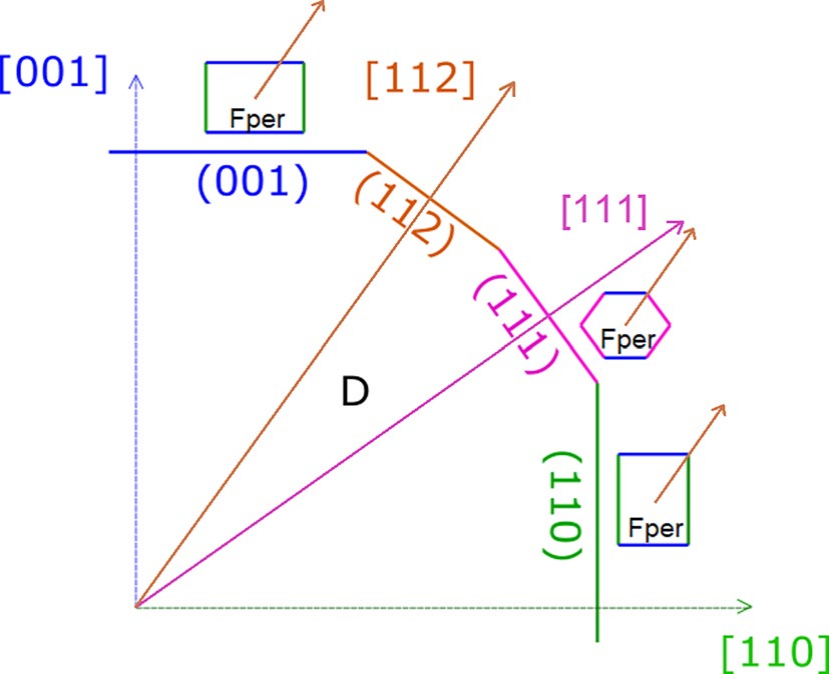
Periclase in epitaxy with diamond. A schematic image representing Fper crystals having the same crystallographic orientation with respect to diamond, ${\langle 100\rangle }_{D}\equiv {\langle 100\rangle }_{Fper}$, but different epitaxial relationships. Blue is for the (001) face of both diamond and periclase, orange is for the (112) face, pink is for the (111) face and green is for the (110) face.

To better explain this concept, let us consider periclase (Fper) inclusions in diamond (D), (Mg,Fe)O (Space Group, S.G.: *F*m$\overline{3}$m), with a specific COR defined by the coincidence of their main crystallographic axes, ${\langle 100\rangle }_{{\text{D}}}\equiv {\langle 100\rangle }_{{\text{Fper}}}$^24,27^. Such specific COR can be realized in several ways (Fig. 1). Indeed, epitaxy between Fper and D can occur through the (001)_D_/(001)_Fper_, (112)_D_/(112)_Fper_, (111)_D_/(111)_Fper_ and (110)_D_/(110)_Fper_ interfaces defined by the 2D-LCs listed in Table 1; all these 2D meshes exhibit very low linear misfit (< 2%), along with moderate 2D cell area misfit (< 3.5%). Consequently, if ${\beta }_{adh}^{ D/Fper}$ estimates are lacking, the most probable among the possible epitaxies is the one with the smallest 2D-LC: (001)_D_/(001)_Fper_.

**Table 1:** 2D cell vectors describing the (001)_D_/(001)_Fper_, (112)_D_/(112)_Fper_, (111)_D_/(111)_Fper_ and (110)_D_/(110)_Fper_ interfaces, calculated using the cell parameter (a_0_) 3.5668 Å^30^ and 4.2121 Å^31^ for diamond and periclase, respectively.

	(001)_D_	(001)_Fper_	Linear and area misfits (%)
Vectors (Å)	6 × [010]_D_ = 21.40 6 × [100]_D_ = 21.40	5 × [010]_Fper_ = 21.06 5 × [100]_Fper_ = 21.06	+ 1.61 + 1.61
Area (Å^2^)	458.56	443.52	+ 3.39
	(112)_D_	(112)_Fper_	Linear and area misfits (%)
Vectors (Å)	6 × [1$\overline{1}$0]_D_ = 30.26 6 × [$\overline{2}$01]_D_ = 47.86	5 × [1$\overline{1}$0]_Fper_ = 29.78 5 × [$\overline{2}$01]_Fper_ = 47.09	+ 1.61 + 1.64
Area (Å^2^)	1448.24	1402.34	+ 3.27
	(111)_D_	(111)_Fper_	Linear and area misfits (%)
Vectors (Å)	6 × [1$\overline{1}$0]_D_ = 30.26 6 × [$\overline{1}$01]_D_ = 30.26	5 × [1$\overline{1}$0]_Fper_ = 29.78 5 × [$\overline{1}$01]_Fper_ = 29.78	+ 1.61 + 1.61
Area (Å^2^)	915.67	886.85	+ 3.25
	(110)_D_	(110)_Fper_	Linear and area misfits (%)
Vectors (Å)	6 × [001]_D_ = 21.40 6 × [1$\overline{1}$0]_D_ = 30.26	5 × [001]_Fper_ = 21.06 5 × [1$\overline{1}$0]_Fper_ = 29.78	+ 1.61 + 1.61
Area (Å^2^)	647.56	627.17	+ 3.25

**Figure 2:**
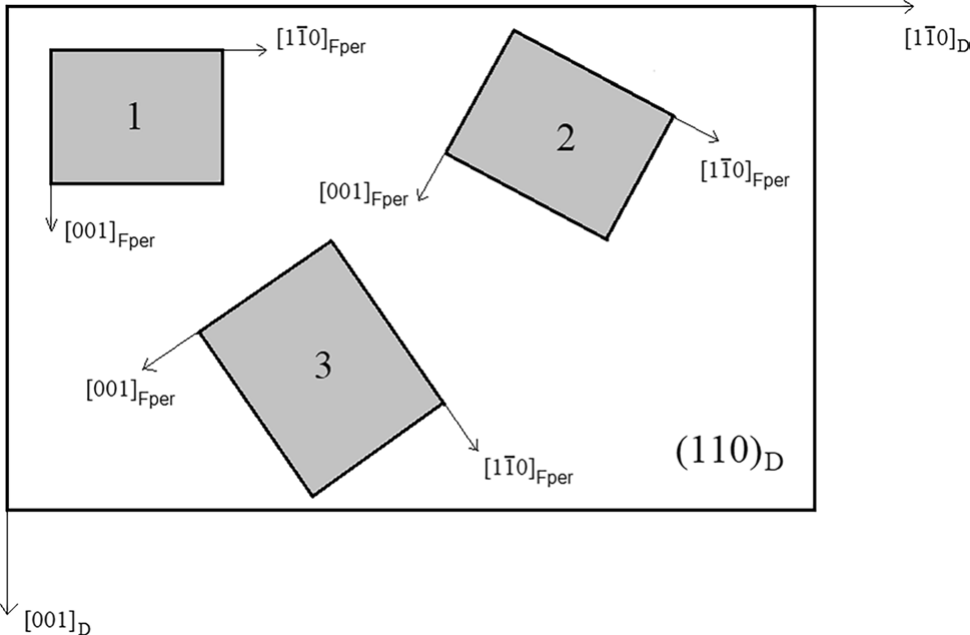
Periclase on diamond showing different crystallographic orientations. A schematic image representing three Fper crystals epitaxially grown with their (110) face above the (110) face of diamond but different 2D coincident meshes (n = 1, 2 and 3 of Table 1). For all the three crystals the direction [110]_Fper_ coincides with [110]_D_.

Furthermore, two crystals can exhibit different crystallographic orientations to each other when in contact along a given interface, because there can be several 2D-LC cells which define the epitaxy. Taking again Fper as an example, it is possible to identify other 2D-LCs at the (110)_D_/(110)_Fper_ interface (2D-LCs, 1 ≤ n ≤ 5 in Table 2). The cell n = 1 has [001]_D_ ≡ [001]_Fper_ and [1$\overline{1}$0]_D_ ≡ [1$\overline{1}$0]_Fper_, the main crystallographic axes of the two crystals coincide, ${\langle 100\rangle }_{{\text{D}}}\equiv {\langle 100\rangle }_{{\text{Fper}}}$. Instead, when considering the other four cells (n = 2, 3, 4 and 5) this is no longer true. They are clockwise rotated with respect to the (n = 1) cell by 30°, 52°, 55° and 23°; the rotation takes place around [110]_D_ ≡ [110]_Fper_. To better clarify what this implies at macroscopic level, in Fig. 2 three Fper crystals epitaxially grown with their (110) face above the (110) face of diamond but different 2D-LCs (n = 1, 2 and 3) are schematized. It follows a high probability to observe Fper crystals rotated at different angles around [110]_Fper_ ≡ [110]_D_, i.e., a rotational statistical COR. Two phases can develop epitaxial relationships when the ${\beta }_{adh}$ value associated with a given interface is high, a requirement that can also occur when the principal axes of the two phases do not coincide.

**Table 2:** Five 2D coincident cells describing epitaxies at the (110)_D_/(110)_Fper_ interface, calculated using the cell parameter (a_0_) 3.5668 Å^30^ and 4.2121 Å^31^ for diamond and periclase, respectively. In the column *Lattice rotation* is reported the angle by which the 2D lattice that defines the (110)_Fper_ is clockwise rotated with respect to the one on the (110)_D_.

n	2D cell	(110)_D_	(110)_Fper_	Linear and area misfits (%)	Lattice rotation (°)
1	Vectors (Å)	6 × [001]_D_ = 21.40 6 × [1$\overline{1}$0]_D_ = 30.26	5 × [001]_Fper_ = 21.06 5 × [1$\overline{1}$0]_Fper_ = 29.78	+ 1.61 + 1.61	0
	Area (Å^2^)	647.56	627.17	+ 3.25	
2	Vectors (Å)	[$\overline{1 }11$]_D_ = 6.18 [$1\overline{1 }3$]_D_ = 8.74	[1$\overline{1 }$0]_Fper_ = 5.96 2 × [001]_Fper_ = 8.42	+ 3.69 + 3.80	30
	Area (Å^2^)	53.98	50.18	+ 7.57	
3	Vectors (Å)	2 × [001]_D_ = 7.13 2 × [$1\overline{1 }1$]_D_ = 12.36	[1$\overline{1 }\overline{1 }$]_Fper_ = 7.30 3 × [001]_Fper_ = 12.64	− 2.33 − 2.22	52
	Area (Å^2^)	71.97	75.34	− 4.47	
4	Vectors (Å)	2 × [$\overline{1 }12$]_D_ = 17.47 2 × [$1\overline{1 }0$]_D_ = 10.09	3 × [1$\overline{1 }$0]_Fper_ = 17.87 [1$\overline{1 }2$]_Fper_ = 10.32	− 2.24 − 2.23	55
	Area (Å^2^)	143.95	153.45	− 6.19	
5	Vectors (Å)	3 × [$1\overline{1 }\overline{1 }$]_D_ = 18.53 4 × [001]_D_ = 14.27	[3$\overline{3 }\overline{1 }$]_Fper_ = 18.36 [$\overline{1 }$13]_Fper_ = 13.97	+ 0.93 + 2.15	23
	Cell area (Å^2^)	215.92	200.72	+ 7.57

## Periclase inclusions in diamond

[(Mg,Fe)O] periclase (Fper) is the most abundant inclusion in super-deep diamonds (those diamonds formed at great depth down to the lower mantle^32^). Its chemical composition varies from nearly pure MgO (periclase) to FeO (wüstite) with X_FeO_ (FeO molar fraction) varying between 0.06 and 0.85^33^. Fper represents ∼42% of the inclusions described within super-deep diamonds. Such abundance does not reflect laboratory experiments, which typically show that Fper accounts for ∼17% of the mantle phase assembly in a mantle bulk composition^34,35^. Moreover, experiments predict that the chemical composition of lower-mantle Fper should be Mg-rich (X_FeO_ between about 0.08 and 0.18^35^), while time to time this mineral in diamond shows a different composition with very high Fe contents^33^. According to some authors, such discrepancies are due to the existence of a non-pyrolitic source in the lower mantle^36,37^. Liu^38^ proposed that Fe-rich Fper and diamond can simultaneously precipitate in the lower mantle, through decarbonation of (Mg,Fe)CO_3_. Thomson et al.^39^ showed that Fper with variable Fe contents and diamond can crystallize simultaneously by interaction between mantle peridotite and slab-derived carbonatite melts in the deep upper mantle or transition zone. In order to investigate the possible genetic processes that formed periclase-bearing diamonds, Nimis et al.^24^ determined CORs for nine Fe-rich Fper inclusions (X_FeO_ between 0.33 and 0.64) in two diamonds. They found that these inclusions are specifically oriented with their diamond hosts, ${\langle 100\rangle }_{{\text{D}}}\equiv {\langle 100\rangle }_{{\text{Fper}}}$, suggesting an epitaxial relationship between the two phases. Accordingly, they proposed that such Fper nucleated during the growth history of the diamond: then, Fe-rich Fper inclusions should be considered syngenetic.

In a more recent work, Lorenzon et al.^27^ determined the CORs for 57 Fper inclusions in 37 diamonds spanning a large compositional range to determine possible associations between the Fper Fe-content and the depth origins of periclase-bearing diamonds. To increase the statistical significance of their analysis, two Fper inclusions (X_FeO_ ≈ 0.40) in a diamond previously studied by Anzolini et al.^40^ were also considered. Interestingly, they found that:

1. 16 inclusions are specifically oriented with their diamond hosts, ${\langle 100\rangle }_{{\text{D}}}\equiv {\langle 100\rangle }_{{\text{Fper}}}$; the same specific orientation reported by Nimis et al.^24^ All these inclusions are thus interpreted to have been specifically oriented at the time of their incorporation.
2. 9 inclusions show a rotational statistical COR, with [110]_D_ ≡ [110]_Fper_ and the other crystallographic directions randomly rotated around this axis.
3. The remaining 32 inclusions do not show any particular crystallographic orientations with respect to their hosts.
4. Within four diamonds there are several iso-oriented inclusions, but their orientation varies in the just-quoted diamonds (i.e., random CORs).
5. Especially, the analysis of inclusions for which both CORs and chemical data were available highlighted a strong correlation between Fe content and crystallographic orientation:

(i) 12 out of 13 Fe-rich Fper inclusions (X_FeO_ > 0.3) have a specific COR, ${\langle 100\rangle }_{{\text{D}}}\equiv {\langle 100\rangle }_{{\text{Fper}}}$;

(ii) 4 out of 5 Mg-rich Fper inclusions (X_FeO_ ≤ 0.2) present random CORs, while the remaining one is compatible with both a random and a rotational statistical COR.

On the basis of these just quoted observations, Lorenzon et al.^27^ suggested a dual origin with two distinct processes to explain the formation of Fe-rich and Fe-poor Fper inclusions:

1. Fe-rich Fper inclusions (X_FeO_ > 0.3) frequently presenting a specific COR, could be considered syngenetic with their diamond hosts and were formed in the deep upper mantle or transition zone. Their composition does not reflect the expected lower mantle composition.
2. Mg-rich Fper inclusions showing random CORs, represent parts of pre-existing mineral assemblages partially dissolved and passively entrapped by diamond during its precipitation in the lower mantle (i.e., protogenetic inclusions) and represent the expected composition from the laboratory experiments.

Although we agree with Lorenzon et al.^27^ on the dual origin of Mg-rich and Fe-rich periclases in terms of depth of formation (lower mantle versus upper mantle and transition zone, respectively), we disagree with the conclusions of Nimis et al.^24^ and Lorenzon et al.^27^ as there are some evidences/observations that led us to suppose that also Fe-rich Fper inclusions (X_FeO_ > 0.3) can be considered protogenetic:

1. many Fe-rich Fper inclusions exhibit lobed morphologies with rounded shapes and/or embayment, so witnessing clear evidence that the mineral undergone dissolution. This is particularly evident in Fig. 1 of Nimis et al.^24^ (and Fig. 1 in Agrosì et al.^41^). The same identical reabsorbed morphology was observed in silicate inclusions, for which their protogenetic origin was demonstrated^2,12,14,15^ (Supplementary Information);
2. a further feature shown in Fig. 1 of Nimis et al.^24^ is that iso-oriented inclusions of Fe-rich Fper are strongly clustered in a portion of the diamond. If these inclusions were syngenetic we could expect that they should be also arranged homogeneously over the diamond and not so grouped. Thus, we cannot exclude that these grouped inclusions are the remaining fragments of a pre-existing single Fper before the dissolution process occurred and that this pre-existing Fper acted as diamond-growth substrate;
3. clustered iso-oriented Fe-rich Fper inclusions are located in proximity of the central growth zone of the diamond (see again Fig. 1E in Nimis et al.^24^). Syngenetic inclusions should be also found in other growth sectors of the diamond (i.e., core and rim) and not located exclusively in the central growth zone;
4. according to the classical nucleation theory, it is highly unlikely to observe the nucleation of such a limited number of crystalline individuals (i.e., Fper) in epitaxy with another phase (i.e., D) during different growth stages of the latter, thus at different times in the diamond's growth history. It is hard to explain, from a chemical-physics point of view, both a so low nucleation frequency and the newly forming minerals always with the same orientation with respect to diamond;
5. finally, imaging that diamond is formed from a carbon-rich fluid or melt, it is reasonable to assume this fluid (or melt) simultaneously supersaturated in the diamond and unsaturated in all the other phases (e.g., silicates, sulphides and oxides). It is conceivable that the fluid percolating in the rock is dynamic (i.e., not static) and consequently remains supersaturated in the diamond and unsaturated in all other phases for the time necessary to the formation of diamonds. It is therefore unlikely that during the growth history of the diamond, this fluid (or melt) will sporadically become supersaturated in periclase to allow its nucleation. Nimis et al.^42^ detected the presence of a fluid in some sectors of the inclusion/diamond interface. This fluid, whose chemical composition is only partly known, could be the one responsible for the diamond formation and dissolution of the subsequently trapped minerals.

**Figure 3:**
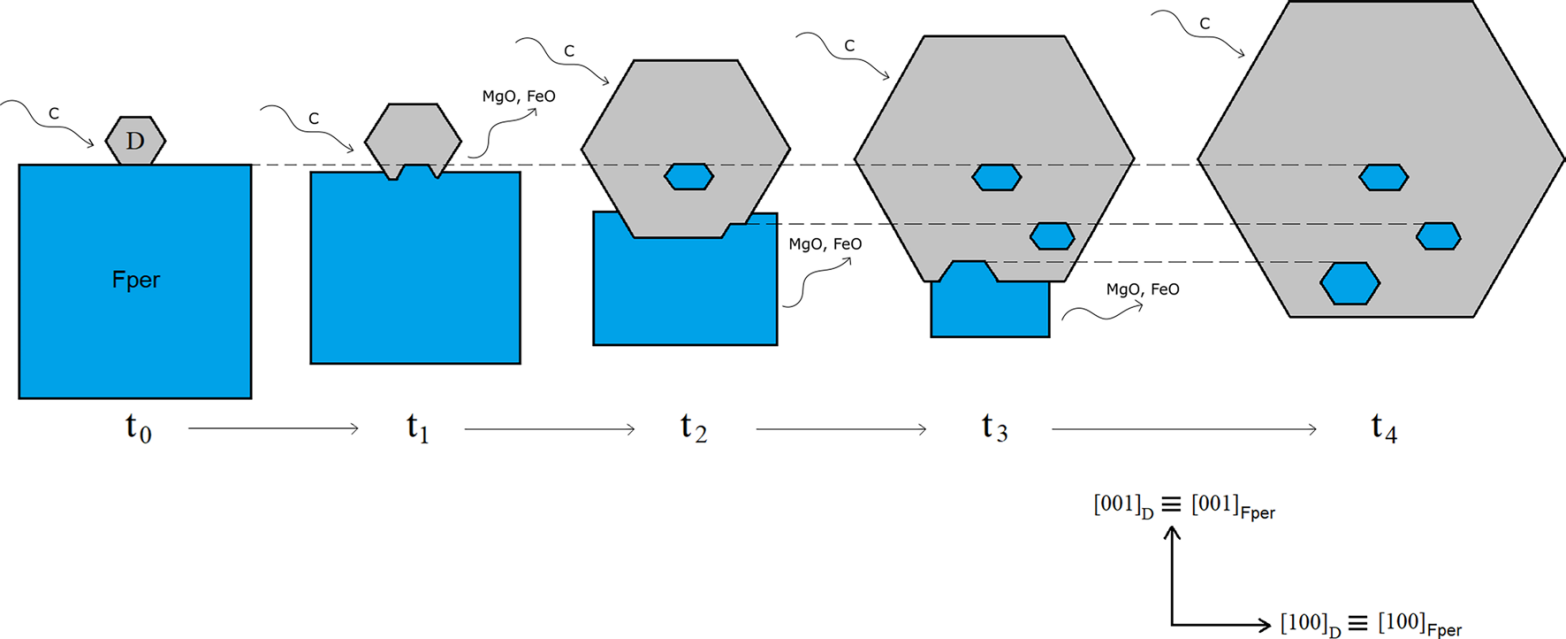
A conceptual model for the growth of diamond (D) in epitaxy with Fe-rich periclase (Fper). At t_0_, a diamond nucleates on a crystal face of periclase, with orientation ${\langle 100\rangle }_{{\text{D}}}\equiv {\langle 100\rangle }_{{\text{Fper}}}$; this specific COR is favoured by a high adhesion energy between the two phases along a specific interface, e.g., (001)_D_/(001)_Fper_. Successively (t_1_ − t_4_), partial dissolution of periclase during diamond growth results in the complete entrapment of several relicts of the original periclase with the same crystallographic orientation. The metasomatic fluid supplying C and dissolving periclase is represented by black arrows.

Based on these evidences/observations, we propose an alternative explanation for the formation of specifically iso-oriented Fe-rich Fper inclusions in diamond. Our model can explain all the characteristics of the inclusions described above, as well as to overcome the inconsistencies found when they are supposed to be syngenetic. When the iron content exceeds a certain limit (X_FeO_ ≥ 0.30), Fper can trigger the heterogeneous nucleation of diamond above one of its crystallographic forms: it is likely that there is a high adhesion energy related to a specific diamond/periclase interface [e.g., (001)_D_/(001)_Fper_] and a 2D coincidence cell (e.g., 6 × [010]_D_ $\equiv$ 5 × [010]_Fper_, 6 × [100]_D_ $\equiv$ 5 × [100]_Fper_; see Table 1), thus determining the observed specific COR, ${\langle 100\rangle }_{{\text{D}}}\equiv {\langle 100\rangle }_{{\text{Fper}}}$. To validate this hypothesis, ab initio calculations of the adhesion energies between diamond and Fe-rich Fper should be performed. After its nucleation time (t_0_ in Fig. 3), diamond starts growing while periclase is dissolving (t_1_ in Fig. 3), since the carbon-rich fluid percolating through the mantle rock is simultaneously supersaturated (in diamond) and unsaturated (in Fper). Partial dissolution of periclase during diamond growth (t_2_-t_4_ in Fig. 3) can result in the complete entrapment of several relicts of the original Fper, such relicts showing the same crystallographic orientation.

This model assumes a protogenetic origin of the Fe-rich Fper inclusions, in analogy with the inclusions of silicates and sulphides (Supplementary Information), but with the difference that in this case the nucleation of the diamond is triggered by the phase itself which will form the iso-oriented inclusions. Moreover, this model also assumes that post-entrapment modification of the inclusions shape cannot occur: Bruno et al.^16^ ruled out that an epitaxial relationship can develop after trapping of inclusions.

We could apply this growth mechanism to the recent work by Lorenzon et al.^27^; thus, while we consider still valid the dual origin of Mg-rich (lower mantle) against the Fe-rich (upper mantle and transition zone) periclases, we suggest that Fper inclusions in diamonds could be all protogenetic. Therefore, on one side we agree with Lorenzon et al.^27^ proposing that Mg-rich periclases are passively entrapped by diamond within the lower mantle; on the other side, we are definitely convinced that Fe-rich Fper are likely the best candidate as diamond growth substrates in the upper mantle and/or transition zone related to subduction processes (in agreement with Thomson et al.^39^).

It is also possible that Fe-rich Fper reacts with the C-rich fluid that percolates the rock to generate, e.g., an epitaxially related coronitic magnesioferrite (MgFe_2_^3+^O_4_) and that the latter subsequently acts as a substrate for the heterogeneous nucleation of diamond, similarly to what was observed for sulphides^21^. Fe^2+^ in periclase oxidizes in Fe^3+^, while, e.g., carbonate ions CO_3_^2−^ in C-rich fluid reduces to produce diamond. Obviously, to verify the existence of a coronitic phase, a detailed TEM study is needed. Interestingly, nanometer-sized Mg-ferrite at the Fper/diamond interface was observed^43^.

Our model agrees with the strong Fe-variability in Fper inclusions in diamond and, at the same time, explains why we have such large amount of Fper inclusions in diamonds (e.g., 42% against the expected 17% from laboratory experiments). We should also remark that the protogenetic model would also support the hypothesis that the number of Fper inclusions in diamond so far reported in literature, does not reflect the real abundance of the phase in the mantle: for instance, when we found four different grouped Fper inclusions in one single diamond we should count them as one single original inclusion and not as four separated inclusions; this would also affect the current running number of Fper inclusions found so far in super-deep diamonds.

Similar considerations were made for magnesiochromite (MgCr_2_O_4_) inclusions (Supplementary Information). We suggest that the majority of magnesiochromite inclusions in diamonds are protogenetic and have been incorporated into the diamond according to the model described above for Fper.

## Conclusions and implications

We critically discussed the universally adopted epitaxial criterion (EC) for determining whether a mineral inclusion is syngenetic or protogenetic with respect to diamond. By revisiting previously published data on periclase and magnesiochromite inclusions in diamond (i.e. their crystallographic orientation, morphology and location within the diamond), we show that despite the existence of epitaxial relationships between inclusions and diamond (i.e. specific CORs), it is not possible to state with certainty that they are syngenetic. Therefore, we suggest abandoning the EC to discriminate between syngenesis and protogenesis. At the current state of our knowledge, EC is not admissible to establish whether an inclusion is syngenetic or protogenetic.

Based on this consideration and reinterpreting the available data through the crystal growth theory, we hypothesize that both Fe-rich periclase (X_FeO_ ≥ 0.30) and magnesiochromite inclusions in diamond are protogenetic: they act as a substrate to allow heterogeneous nucleation of diamond with specific or rotational CORs.

Combining our observations on periclase and magnesiochromite inclusions with previous works that have already demonstrated the protogenetic origin of silicate and sulphide inclusions, we advance the hypothesis that the majority of inclusions in diamonds are protogenetic, i.e., they are relicts of dissolving single crystals. This would mean that the majority of inclusions are constituent minerals of the rock in which diamonds are formed and not products of reactions that occur during diamond growth. However, it is reasonable to expect that the dissolving phases can modify their composition (major and trace elements) through exchange reactions with the fluid supersaturated in diamond and percolating into the rock.

Finally, the protogenetic model we propose supports the hypothesis that the number of inclusions in diamonds so far reported in literature, needs to be revised: grouped and iso-oriented inclusions in one single diamond should count as one single original inclusion and not as separated inclusions. Statistics are further distorted for minerals favouring the nucleation of diamonds. The latter will be forced to incorporate the minerals on which they were formed. Hence, it would be necessary to completely review the statistics on inclusions found in diamonds.

### Supplementary Information

Supplementary Information.

## Data Availability

All data generated or analysed during this study are included in this published article.
