# Supplementary material for: A critique of using epitaxial criterion to discriminate between protogenetic and syngenetic mineral inclusions in diamond
Source: Sci Rep. 2024 Apr 15;14:8674. doi: 10.1038/s41598-024-59432-6 (PMC11018738; doi:10.1038/s41598-024-59432-6)
Supplement: Supplementary file 1 — Supplementary Information. [file 41598_2024_59432_MOESM1_ESM.docx]

**A critique of using epitaxial criterion to discriminate between protogenetic and syngenetic mineral inclusions in diamond**

Marco BRUNO,^1,2^ Stefano GHIGNONE,^1^ Dino AQUILANO,^1^ Fabrizio NESTOLA,^3^*

^1^ Dipartimento di Scienze della Terra, Università degli Studi di Torino, Via Valperga Caluso 35, 10125, Torino (TO), Italy

^2^ NIS, Centre for Nanostructured Interfaces and Surfaces, Università degli Studi di Torino, Via G. Quarello 15/a, 10135, Torino (TO), Italy

^3^ Dipartimento di Geoscienze, Università degli Studi di Padova, Via Gradenigo 6, I-35131 Padova, Italy

*Corresponding author

e-mail: [fabrizio.nestola@unipd.it](mailto:fabrizio.nestola@unipd.it)

**SUPPLEMENTARY INFORMATION**

**Contents**

1. Silicates and sulphides inclusions in diamond
2. Magnesiochromite (ideally ~ MgCr_2_O_4_) inclusions in diamond

**1. Silicates and sulphides inclusions in diamond**

Silicates and sulphides are the most common lithospheric inclusions in diamonds^1^: 32% are garnet, [(Mg,Fe,Ca)_3_(Al,Cr)_2_Si_3_O_12_]; 16% are olivine, [(Mg,Fe)_2_SiO_4_]; 16% are Cr-spinel, [(Mg,Fe)(Cr,Fe,Al)_2_O_4_]; 15% sulphides, primarily pyrrhotite (FeS) with subordinate pentlandite [(Fe,Ni)_9_S_8_] and chalcopyrite (CuFeS_2_); 13% clinopyroxene, [(Ca,Na,Mg,Fe,Al)_2_Si_2_O_6_]; 7% orthopyroxene [(Mg,Fe,Ca)_2_Si_2_O_6_]; 1% rutile, (TiO_2_); 1% coesite, (SiO_2_).

To provide statistically significant data on the relative crystallographic orientations of olivine inclusions in diamond, Nestola et al.^2^ and Milani et al.^3^ determined the orientations of 63 olivine inclusions with both diamond-imposed and lobate morphology in 28 different diamonds from the Udachnaya kimberlite in Siberia (Russia) and from Bultfontein, Koffiefontein and Premier kimberlites from the Kaapvaal craton (South Africa). No overall preferred orientation of these olivines in diamond were detected (i.e., random CORs). However, multiple inclusions within a single diamond frequently exhibit similar orientations, implying that they were derived from original single pre-existing olivine monocrystals (i.e., protogenetic olivines).

Nestola et al.^4^ investigated 30 garnet inclusions in 20 diamonds (Udachnaya, Siberia; Jericho, Canada; Voorspoed, South Africa; Jwaneng and Letlhakane, Botswana; Meratus Mountains, southeast Borneo): 11 eclogitic and 9 peridotitic diamonds were examined. Garnets (also with pseudo cubo-octahedral and lobate shapes) showed no specific COR with respect to the diamonds, which excludes any epitaxial relationships with their hosts. Even in this case, multiple garnet inclusions within a single diamond exhibit similar orientations, suggesting that such inclusions are the remains of a single pre-existing crystal that was partially dissolved during or before diamond growth (i.e., protogenetic garnets). Moreover, the authors observed the breadth of the iso-oriented garnet clusters is consistent with the typical crystal size of garnet in mantle rocks.

Nestola et al.^5^ discovered in a diamond-bearing peridotite a clinopyroxene (external to diamond) with the same composition and crystal orientation of the clinopyroxene relicts included in the diamond. This finding indicates that such clinopyroxene inclusions are definitively protogenetic.

Pamato et al.^6^ investigated 17 pyrrhotite inclusions in 12 diamonds (Victor kimberlite and Jericho kimberlite, Canada). The authors observed that 14 of the 17 sulphide inclusions had random CORs, while just three had a crystallographic axis coincident with one of its host, suggesting the absence of crystal growth control between the diamond and inclusions. Moreover, similarly to what was observed for the silicates described above, multiple pyrrhotite inclusions within a single diamond have similar orientations, suggesting that these inclusions are remains of a pre-existing, partially dissolved single crystal (i.e., protogenetic sulphides). A previous isotopic study performed by Thomassot et al.^7^ also suggests a protogenetic origin for sulphides inclusions in diamonds. Finally, Jacob et al.^8^ established a protogenetic origin for pyrrhotite inclusions in diamond by analysing their plastic deformation features. In addition, they also observed a corona of nanocrystalline magnetite (Fe_3_O_4_) surrounding a crystal of pyrrhotite. This corona shows epitaxy between all three phases (pyrrhotite, magnetite and diamond) with the crystallographic <111> axes of magnetite aligned with the <100> axes of both diamond and pyrrhotite. According to the authors, the epitaxy indicates that the coronitic magnetite was formed at the expense of sulphide, after which diamond nucleated on the surface of magnetite and eventually encasing the assemblage and terminating the redox reaction responsible for the diamond formation.

**2. Magnesiochromite (ideally ~ MgCr_2_O_4_) inclusions in diamond**

Viggers de Vries et al.^9^ identified a specific COR for five magnesiochromite (hereafter CHR) inclusions in a diamond from Udachnaya (Russia): $\left\langle100 \right\rangle_{D}\equiv\left\langle100 \right\rangle_{\mathrm{CHR}}$. The inclusions were located in the core-intermediate diamond growth sectors. Based on the similar crystallographic orientation of the host diamond and the chromite inclusions, the authors concluded that the diamond host controlled the orientations of the inclusions, and hence, a syngenetic origin for the inclusions was supposed.

The same crystallographic orientation was previously reported by Frank-Kamenetsky^10^ for 4 out of 9 CHR inclusions in 9 diamonds studied. Of the remaining five inclusions, four show a different specific COR, that is [111]_D_ $\equiv$ [111]_CHR_ and [101]_D_ $\equiv$ [112]_CHR_.

More recently, Nimis et al.^11^ investigated the CORs of 36 CHR inclusions in 23 different diamonds from three localities (Udachnaya, Russia; Damtshaa, Botswana; Panda, Canada). These authors observed that:

1. for 17 inclusions out of 36 (47%): [111]_D_ $\equiv$ [111]_CHR_. In particular, for 6 out of 17 inclusions, [1$\overline{1}$0]_D_ $\equiv$ [1$\overline{1}$0]_CHR_ axis (the angle between these two directions was lower than 4°), for 5 inclusions [1$\overline{1}$0]_D_ ≈ [1$\overline{1}$0]_CHR_ (~6° < [1$\overline{1}$0]_D_ ^^^ [1$\overline{1}$0]_CHR_ < ~11°), while the remnants 6 were variously rotated around the [111] axis. Therefore, for the majority (11) of these 17 inclusions it has been determined the following crystallographic orientation: $\left\langle100 \right\rangle_{D}\equiv\left\langle100 \right\rangle_{\mathrm{CHR}}$, the same already found by Viggers de Vries et al.^9^ and Frank-Kamenetsky^10^. Nimis et al.^11^ classified these 17 inclusions as a case of rotational statistical COR. The remaining 19 CHR inclusions (53%) had a random COR with diamond. Therefore, two groups of data were identified by Nimis et al.^11^: one group characterized by rotational statistical COR around [111] (47% of pairs) and one group with random COR (53% of pairs).
2. In most cases, the inclusions appeared to be located at the periphery of the diamonds. Anyway, as widely discussed in Bruno et al.^12^, these data can be misleading when they are not associated to specific growth sectors observed by cathodoluminescence; we must remark that any attempted localization of an inclusion in diamond only by optical observations can be completely wrong. Indeed, only when diamond grows homothetically (i.e., equivalent faces advance with the same rate), the crystal gravity-centre coincides with the central growth zone of the diamond. When the growth is not homothetic, then the central growth zone of the diamond no longer coincides with the gravity-centre of the crystal. This means that an inclusion apparently located on the periphery of the diamond may actually be in the central growth zone of the diamond itself.
3. Nimis et al.^13^ detected the presence of a fluid film around most of the inclusions studied in Nimis et al.^11^. However, as specified by the authors, the resolution of the analytical technique adopted is insufficient to prove that the fluid continuously surrounds the inclusions. According to the authors, the detection of a fluid suggests a low adhesion between inclusion and diamond, thus excluding an epitaxial relationship.
4. Iso-oriented multiple inclusions were found within single diamonds (three diamonds: Oli_CHR1, MgCr_4 and BOTS_01). In particular, the inclusions in Oli_CHR1 have orientation $\left\langle100 \right\rangle_{D}\equiv\left\langle100 \right\rangle_{\mathrm{CHR}}$, those in MgCr_4 have [111]_D_ $\equiv$ [111]_CHR_ and those in BOTS_01 are differently oriented with respect to those in the other two diamonds. The authors interpreted these iso-oriented inclusions as portions of a pre-existing monocrystal that was partially dissolved during the formation of the diamond. This is the same interpretation given by Nestola et al.^2^ for some multiple olivine inclusions in diamonds, which are interpreted as protogenetic.

On the basis of these observations, Nimis et al.^11^ suggested that iso-oriented inclusions in Oli_CHR1, MgCr_4 and BOTS_01 are protogenetic, whereas for the inclusions showing rotational statistical COR is not possible to establish their origin. Indeed, the authors wrote that such rotational COR is due to “*mechanical interactions between octahedral crystals in a fluid-rich environment before final incorporation, regardless of the proto- or syngenetic nature of the inclusions*”. They suppose a mechanical juxtaposition between diamond and chromite at the (111)_D_/(111)_CHR_ interface. We disagree with the conclusions drawn by Viggers de Vries et al.^9^ and Nimis et al.^11^. Instead, we believe that all the magnesiochromite inclusions are protogenetic and that several epi-relationships develop between the two phases along the (111)_D_/(111)_CHR_ interface. Indeed, the orientation $\left\langle100 \right\rangle_{D}\equiv\left\langle100 \right\rangle_{\mathrm{CHR}}$ observed by all authors^9-11^ is difficult to justify with the mechanical juxtaposition model proposed by Nimis et al.^11^; it would be certainly more reliable, from a chemical-physics point of view, to assume a good adhesion between the phases along the (111)_D_/(111)_CHR_ interface, for which it is possible to identify several 2D-LCs (Table S1) with low linear and areal misfits (< 6%). By analysing Table S1 the 2D coincidence cell with lowest linear and areal misfits (< 1%) is the n = 1, the one for which the main crystallographic axes of the two crystals coincide. Moreover, the other 2D-LCs are rotated with respect to the cell n = 1 by 9°, 15°, 45° and 90° (the rotation takes place around [111]_D_ ≡ [110]_CHR_), thus it is reasonable to assume that the rotational statistical COR described by Nimis et al.^11^ is a consequence of several epi-relationships occurring at the (111)_D_/(111)_CHR_ interface. Although there are no estimates of interfacial energies, the existence of several good 2D-LCs leads us to hypothesize a strong affinity between diamond and magnesiochromite, and that therefore the fluid does not completely surround the inclusions. If, in addition we also consider that several inclusions are the result of a dissolution of an original single crystal, we state that all magnesiochromite inclusions in diamonds are protogenetic and have been incorporated into the diamond according to the model previously described for periclase inclusions. It is likely that the different 2D-LCs described in Table S1 have high and very similar adhesion energies, favouring in this way the heterogeneous nucleation of diamonds differently oriented above the (111) _CHR_ and thus determining the observed rotational statistical COR described by Nimis et al.^11^

As for periclase, if also all magnesiochromites are protogenetic, then this would strongly affect the general numbering of these inclusions and would explain why magnesiochromites are so abundant in lithospheric diamonds (about 16% of all inclusions^1^). More in detail:

1. if many separated inclusions were from the same pre-existing mineral (protogenetic model), then we should count only one inclusion instead of counting all fragments belonging to that pre-existing grain;
2. at the same time, we could not exclude that magnesiochromite could act as diamond growth triggering substrate.

Options 1) and 2) do not exclude each other and if combined would really justify the overabundance of this spinel mineral in lithospheric diamonds.

**Table S1.** Five 2D coincident cells describing epitaxies at the (111)_D_/(111)_CHM_ interface, calculated using the cell parameter (a_0_) 3.5668 Å^14^ and 8.3342 Å^15^ for diamond and magnesiochromite, respectively. In the column *Lattice rotation* is reported the angle by which the 2D lattice that defines the (111)_CHM_ is anti-clockwise rotated with respect to the one on the (111)_D_.

| **n** | **2D cell** | **(111)_D_** | **(111)_CHM_** | **Linear and area misfits (%)** | **Lattice rotation (°)** |
| --- | --- | --- | --- | --- | --- |
| 1 | vectors (Å) | 7×[1$\bar{1}$0] = 35.31  7×[$\bar{1}$01] = 35.31 | 3×[1$\bar{1}$0] = 35.37  3×[$\bar{1}$01] = 35.37 | -0.17  -0.17 |  |
|  | area (Å^2^) | 1246.80 | 1251.04 | -0.34 | 0 |
|  |  |  |  |  |  |
| 2 | vectors (Å) | [5$\bar{41}$] = 23.12  [$\bar{6}$15] = 23.12 | 2×[1$\bar{1}$0] = 23.58  2×[$\bar{1}$01] = 23.58 | -1.95  -1.95 |  |
|  | area (Å^2^) | 534.53 | 556.02 | -3.86 | 9 |
|  |  |  |  |  |  |
| 3 | vectors (Å) | 2×[2$\bar{3}$1] = 36.38  2×[$\bar{3}\bar{1}$4] = 36.38 | 3×[1$\bar{1}$0] = 35.37  3×[$\bar{1}$01] = 35.37 | +2.86  +2.86 |  |
|  | area (Å^2^) | 1323.50 | 1251.04 | +5.79 | 15 |
|  |  |  |  |  |  |
| 4 | vectors (Å) | 2×[4$\bar{1}\bar{3}$] = 36.38  2×[1$\bar{3}2$] = 36.38 | 3×[1$\bar{1}$0] = 35.37  3×[$\bar{1}$01] = 35.37 | +2.86  +2.86 |  |
|  | area (Å^2^) | 1323.50 | 1251.04 | +5.79 | 45 |
|  |  |  |  |  |  |
| 5 | vectors (Å) | 4×[1$\bar{1}$0] = 20.18  4×[01$\bar{1}$] = 20.18 | [$\bar{11}$2] =20.42  [$\bar{2}$11] =20.42 | -1.18  -1.18 |  |
|  | area (Å^2^) | 407.23 | 416.98 | -2.34 | 90 |

**References**

1. Stachel, T. & Harris, J. W. The Origin of Cratonic Diamonds—Constraints from Mineral Inclusions. *Ore Geol. Rev.* **34**, 5– 32 (2008).

2. Nestola, F., Nimis, P., Angel, R.J., Milani, S., Bruno, M., Prencipe, M. & Harris, J.W. Olivine with diamond-imposed morphology included in diamonds: Syngenesis or protogenesis? *Int. Geol. Rev.* **56**, 1658–1667 (2014).

3. Milani, S., Nestola, F., Angel, R.J., Nimis, P. & Harris, J.W. Crystallographic orientations of olivine inclusions in diamonds. *Lithos* **265**, 312–316 (2016).

4. Nestola, F., Jacob, D.E., Pamato, M.G., Pasqualetto, L., Oliveira, B., Greene, S., Perritt, S., Chinn, I., Milani, S., Kueter, N., Sgreva, N., Nimis, P., Secco, L. & Harris, J.W. Protogenetic garnet inclusions and the age of diamonds. *Geology* **47**, 431–434 (2019).

5. Nestola, F., Jung, H. & Taylor, L.A. Mineral inclusions in diamonds may be synchronous but not syngenetic. *Nat. Commun.* **8**, 14168 (2017).

6. Pamato, M.G., Novella, D., Jacob, D.E., Oliveira, B., Pearson, D.G., Greene, S., Afonso, J.C., Favero, M., Stachel, T., Alvaro, M. & Nestola, F. Protogenetic sulfide inclusions in diamonds date the diamond formation event using Re-Os isotopes. *Geology* **49**, 941–945 (2021).

7. Thomassot, E., Cartigny, P., Harris, J.W., Lorand, J.P., Rollion-Bard, C. & Chaussidon, M. Metasomatic diamond growth: A multi-isotope study (^13^C, ^15^N, ^33^S, ^34^S) of sulphide inclusions and their host diamonds from Jwaneng (Botswana). *Earth Planet. Sci. Lett.* **282**, 79–90 (2009).

8. Jacob, D.E., Piazolo, S., Schreiber, A. & Trimby, P. Redox-freezing and nucleation of diamond via magnetite formation in the Earth’s mantle. *Nat. Commun.* **7**, 11891 (2016).

9. Wiggers de Vries, D.F., Drury, M.R., de Winter, D.A.M., Bulanova, G.P., Pearson, D.G. & Davies, G.R., Three-dimensional cathodoluminescence imaging and electron backscatter diffraction: Tools for studying the genetic nature of diamond inclusions. *Contrib. to Mineral. Petrol.* **161**, 565–579 (2011).

10. Frank-Kamenetsky, V.A. *The nature of structural impurities and inclusions in minerals* (Leningrad, Gos. Univ., 1964).

11. Nimis, P., Angel, R.J., Alvaro, M., Nestola, F., Harris, J.W., Casati, N. & Marone, F. Crystallographic orientations of magnesiochromite inclusions in diamonds: What do they tell us? *Contrib. to Mineral. Petrol.* **174**, 29 (2019).

12. Bruno, M., Ghinone, S., Aquilano, D., Nestola, F. Is the imposition of diamond morphology on mineral inclusions a syngenetic or post-genetic process with respect to diamond formation? *Cryst. Growth Des.* **23**, 5279-5288 (2023).

13. Nimis, P., Alvaro, M., Nestola, F., Angel, R.J., Marquardt, K., Rustioni, G., Harris, J.W. & Marone, F. First evidence of hydrous silicic fluid films around solid inclusions in gem-quality diamonds. *Lithos* **260**, 384–389 (2016).

14. Riley, D.P., Lattice constant of diamond and the C-C single bond. *Nature* **153**, 587-588 (1944).

15. O'Neill, H.St.C. & Dollase, W.A. Crystal structures and cation distributions in simple spinels from powder XRD structural refinements: MgCr_2_O_4_, ZnCr_2_O_4_, Fe_3_O_4_ and the temperature dependence of the cation distribution in ZnAl_2_O_4_. *Phys. Chem. Miner.* **20**, 541-555 (1994).
